# Versatility of unilateral propeller groin flap for coverage of large scrotal defects and its impact on testicular function

**DOI:** 10.1016/j.jpra.2022.08.007

**Published:** 2022-09-18

**Authors:** Mohamed T. Younes, Ayman M. Abdelmofeed, Ola Seif, Mohamed H. Abdelhalim

**Affiliations:** Department of General Surgery, Plastic Surgery Unit, Benha Faculty of Medicine, Benha University, Egypt

**Keywords:** Scrotal reconstruction, Groin flap, Propeller flap, Testicular functions

## Abstract

**Background:**

There are many options for scrotal reconstruction, each having its own benefits and drawbacks. In the last years, the introduction of the propeller flaps gained great popularity, while the use of groin flap as propeller in scrotal reconstruction remains anecdotal, with only a few cases reported in the literature.

**Objectives:**

In this research, we study the versatility of unilateral propeller groin flaps in the reconstruction of large scrotal defects.

**Patients and Methods:**

This study was done on 10 patients with scrotal defects who were admitted to the plastic unit of the general surgery department at Benha University Hospital from 2019 to 2021 for scrotal reconstruction.

**Results:**

All surgeries were successful with a mean operative time of 103.5 min, ranging from 90 to 130 min. All flaps survived well with no flap necrosis, and only one case showed flap congestion. Donor site healed well with the scar hidden in a natural crease, with no affection on the testicular functions.

**Conclusions:**

The use of groin flap as a propeller allows for free movement and rotation of the flap, which allows for better coverage of a large scrotal defect with good vascularity, lesser complications, no need for further operations to separate the pedicle, and the lowest donor site morbidity.

**Level of evidence:**

Level IV, therapeutic study

## Introduction

Originally described by McGregor and Jackson in their approach to create a flap for hand defect coverage in 1972, the groin flap is a vascularized axial flap that uses the superficial circumflex iliac artery (SCIA) arising from the femoral artery just below the inguinal ligament, it covers defective tissue with a pedicled flap technique, retaining a broad skin bridge at its base (1). Daniel and Taylor described a free flap version in 1973 (2).

But the McGregor flap has been used for a long time as it offers greater advantages, including a larger surface of skin, easily concealable scar of the donor site, and does not require microsurgery. (3)

The groin flap was also used locally in the advancement flap to reconstruct trochanteric, penile, perineal, or abdominal areas. (4)

In 1991, Hyakusoku et al. were the first to describe the term “propeller flap” as an adipocutaneous flap that is based on a central subcutaneous pedicle, with a shape resembling a propeller rotated 90 degrees.(5)

In 2006, Hallock (6) combined the concepts of both propeller and perforator flaps, which was similar in shape to that of a fasciocutaneous flap described by Hyakusoku but was based on a skeletonized perforating vessel and rotated 180 degrees on an eccentric pivot point.

Teo et al. (7) provided the greatest contribution to the surgical technique and application of the perforator propeller flap.

Within the last couple of years, propeller flaps gained great popularity, especially for its use in surgical reconstruction of defective soft tissue, which has been described by several authors (8, 9).

The benefits of using perforator propeller flaps include: 1- harvesting the flaps is rapid and easy to use, 2- there is no microsurgery required, 3- they provide a reliable vascular pedicle, and 4- they can undergo wide mobilization and rotation. However, to prevent any complications, accurate patient selection, preoperative planning, and proper dissection techniques are mandatory.

Many scrotal defects are as a result of trauma or infection, especially Fournier's gangrene (10), which is an acute polymicrobial rapidly spreading necrotizing fasciitis of the perineal, genital, and perianal regions.

It is predominately discovered in men and sometimes women and is not limited to the scrotal/perineal region, but when it is affected, it results in a significant loss of skin and subcutaneous tissue in terms of management. (11)

Significant debridement may cause varying scrotal skin defects leading to exposure of the testes. The goal of reconstruction is to create an aesthetically acceptable neoscrotum, which is essential for psychological and social rehabilitation of a sexually active male (12).

In major scrotal and perineal defects, local fasciocutaneous flaps provide adequate coverage, avoiding skin graft problems. Medial thigh flap has been described, with anatomical variation based on the deep external pudendal, anterior branch of the obturator, and medial circumflex femoral artery. Anatomical studies of the perineal region and gluteal fold flap have shown nourishment by the internal pudendal artery. (13)

Using a groin flap as propeller in the reconstruction of scrotal defects remains anecdotal, with limited cases reported in the literature. The wide arc of rotation of the groin flap when used as a propeller allows for good coverage of scrotal defects even if it is large; hence, we study here the versatility of unilateral propeller groin flaps in the reconstruction of large scrotal defects.

### Patient and Methods

Ten patients were enrolled in this study. These patients had scrotal defects and were admitted to our plastic unit in the general surgery department of Benha University Hospital from 2019 to 2021 for scrotal reconstruction after obtaining approval from the local ethical committee and after fully informed written consents were taken.

The cause of the scrotal defect was Fournier's gangrene in seven of the 10 patients and trauma in three of the patients. The mean defective area was from 60 to 150 cm^2^. All patients were examined systematically to evaluate, control, and treat any general diseases or life-threatening conditions. Local wound care was done by surgical debridement of all necrotic tissues, and daily dressing applications were done using saline irrigation and povidone iodine. A swab was taken from the wound for culture and sensitivity, and the proper antibiotic treatment was given.

Inclusion criteria were scrotal defect with major skin loss, while exclusion criteria included minor skin loss that can be treated with release and primary closure, and patients with significant co-morbidities, including advanced renal or liver disease, cardiovascular complications, and pulmonary problems that would interfere with the surgery.

Preoperative semen analysis was performed in the biochemistry laboratory of Benha University Hospital.

## Surgical Technique

**Fig. 1:**
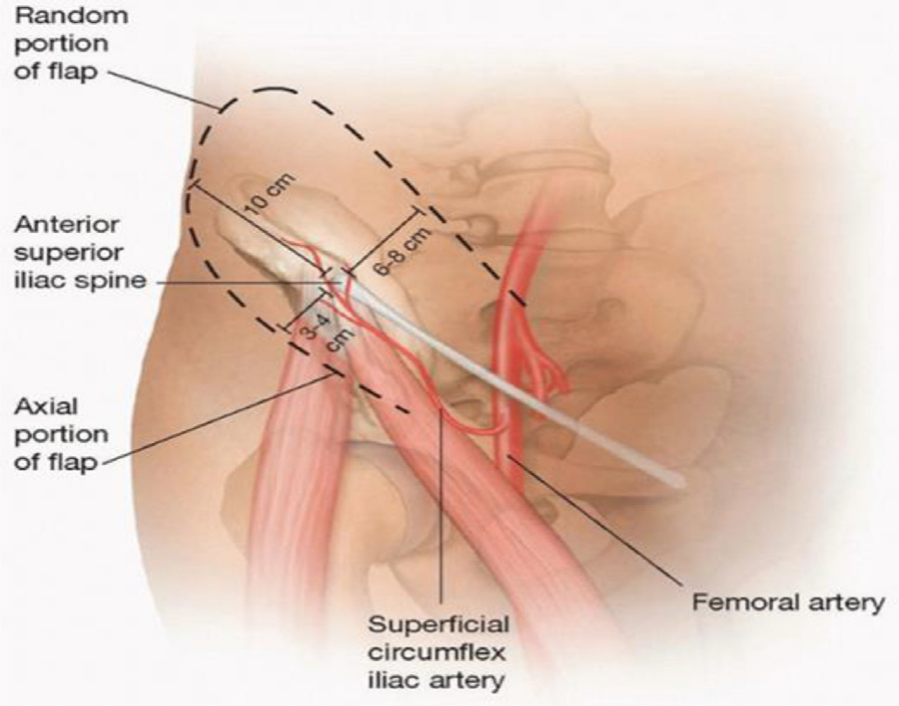
Blood supply of the groin Reprinted from https://plasticsurgerykey.com/pedicled-and-free-groin-flap/Oct 14, 2019 with permission from author Cao Xuan cu

The groin flap is supported by the SCIA. Fig. 1

• The SCIA arises 2 to 3 cm distal to the inguinal ligament, either directly from the femoral artery (70 percent) or from the superficial inferior epigastric artery (30 percent).
• The SCIA crosses laterally from its origin and gives a deep branch at the medial border of the sartorius. After piercing the fascia at the lateral border of the sartorius, the superficial branch continues 2 to 3 cm distal and parallel to the inguinal ligament, toward the anterior superior iliac spine (ASIS).
• The skin for the flap is supplied by this superficial branch. After reaching the ASIS, the superficial branch of the SCIA branches further and anastomoses with branches of the superior gluteal, deep circumflex iliac, and ascending lateral femoral circumflex arteries.
• The flap should be two-third higher than the vascular axis and one-third lower than the axis; this translates to a distance of up to 6 to 7 cm above the axis and 3 to 4 cm below the axis.
• The lateral section of the flap should have a length-to-width ratio of 1:1.This is due to the vascularization pattern on the flap lateral to the ASIS being random. As a result, because the flap's width is limited to 10 cm, the flap's lateral is limited to the ASIS (anterior superior iliac spine).
• The flap's venous drainage is shared between the superficial circumflex iliac vein and the SCIA's venae comitantes.

Eventually, both of them drain into the femoral vein, either directly or through the saphenous vein.

2 Patient position and Graft location

This surgical procedure was performed with all patients in supine position. Important landmarks were marked to determine the flap design including the Sartorius muscle, iliac crest, and inguinal ligament. To determine the exact location of the arterial pedicle (approximately a fingerbreadth below the inguinal ligament), handheld Doppler was used.

3 Flap design, dissection, and harvesting:

Pinching of the skin to assess the tension of closure of the flap after harvesting is done to identify the maximum width of the design. The flap dissection can be started laterally or medially. We preferred to start the incision superiorly and elevate from the distal superior aspect toward the flaps’ medial aspect. The incision was extended downwards to the deep fascia, where the dissection started medially till the level of the ASIS, the interval between the tensor fascia lata and the Sartorius muscle. Structures visualized during the dissection included the perforating vessels, which were ligated and the lateral femoral cutaneous nerve of the thigh as it leaves the deep fascia to enter the subcutaneous tissue. The nerve may require transection depending in its course. After identifying the lateral aspect of sartorius, the muscular fascia is incised along the lateral aspect, and the flap elevation plane is now conducted deep to the muscular fascia. As the dissection continued medially, the superficial circumflex iliac vessels became visible in the plane above the Sartorius heading into the muscular fascia. Skin incisions extended inferiorly and medially to relieve tension in order to avoid transecting the pedicle. All muscular branches were ligated to reduce blood loss. The fascial plane was incised around the pedicle at the medial aspect of the sartorius muscle, and the vessels (artery and vein) were dissected from their origin. The flap was mobilized on the vascular pedicle. The artery may arise either from the femoral vessel/ trunk/ or parent vessel supplying the SCIA and DCIA or may arise from the common trunk that becomes the SIEA (superficial inferior epigastric artery). The vein arises from either the saphenous vein or a branch of the superficial femoral vein. The donor area is closed after slight undermining superficial to the deep fascia. In cases of excessive tension, a layered closure over the suction drains and slight hip flexion were required. The hip can be extended over the course of a few days.

### Postoperative care

After the surgery, patients were sent to the intensive care unit where they were monitored for a short period. For more than five days, the patients remained in supine position with their thighs internally rotated and flexed to relieve any tension on the flap and donor site. Mobility (ex. getting out of bed) was allowed from day one postsurgery. Other postoperative care included giving a course of antibiotics, and any drainage was removed 72 h after the procedure. No anticoagulants were given during or after the surgery. At day five, patients were discharged and followed-up at our outpatient clinics. After 10 days, the interrupted skin sutures were removed. All patients were followed up for a duration of up to one year, and semen analysis was done at 6 and 12 months after the surgery.

## Results

From September 2019 to September 2021, the study was done on 10 patients with large scrotal defects. The median follow-up period was 13 months (range: 12-14 months). The age of patients ranged from 27 to 61 years, with an average age of 46.1 years. The cause of scrotal defect was Fournier gangrene in 7 patients and trauma in 3 patients. Seven of the 10 patients had a comorbidities in the form of hypertension (n=2), diabetes mellitus (n=3), or both (n=2). Four of the ten patients were smokers and were advised to stop smoking two weeks prior to the surgery. Table 1

**Table 1:** Data about the scrotal defect (causes, size, and concomitant disorders)

Case no.	Age	Cause of scrotal defect	Size of the defect	Concomitant disease
1	34	Trauma	70 cm2	No
2	56	Fournier gangrene	64 cm2	DM
3	50	Fournier gangrene	96 cm2	DM and HTN
4	44	Fournier gangrene	105 cm2	DM
5	27	Trauma	67 cm2	DM
6	47	Fournier gangrene	110 cm2	DM and HTN
7	61	Fournier gangrene	88 cm2	DM
8	55	Fournier gangrene	94 cm2	DM and HTN
9	48	Trauma	94 cm2	no
10	39	Fournier gangrene	75 cm2	DM and HTN

Fournier's gangrene cases were treated for 2-3 weeks by serial surgical debridement and dressings. Reconstruction was performed when the local infection subsided, and the wound showed healthy granulation tissue. They were placed on the combination of antibiotics, which included the cephalosporin group, aminoglycoside, and metronidazole. None of these patients showed fungal growth locally or systemically. Blood transfusion was an important part in bringing up the low hemoglobin status in these patients. Local dressing was done with eusol, 1% acetic acid/Silver sulfadiazine.

Regarding the operative details, the length of the designed flap ranged from 12 to 20 cm, with an average of 15.8 cm, the width of the flap ranged from 6 to 9 cm, with an average of 7 cm. The arc of rotation of the flap ranged from 130 to 170 degrees. The flap thickness ranged from 10 to 15 mm, with an average of 12.8 mm. Operative time ranged from 90 to 130 min, with a mean of 103.5 min. Postoperative hospital stay ranged from 4 to 7 days. Table 2

**Table 2:** Operative data about the flap

Case no.	Flap size	Operative time	Arc of rotation of the flap	Flap thickness	Postoperative hospital stay	Flap complications	Donor area complications
1	7*15 cm	95 min	135 degrees	15 mm	5 days	No	No
2	6*14 cm	100 min	150 degrees	12 mm	6 days	No	No
3	9*16 cm	105 min	165 degrees	10 mm	4 days	No	No
4	8*20 cm	120 min	155 degrees	15 mm	7 days	Wound infection	No
5	6*13 cm	90 min	130 degrees	14 mm	5 days	No	No
6	7*19 cm	110 min	160 degrees	12 mm	6 days	No	No
7	7*18 cm	100 min	170 degrees	10 mm	5 days	No	No
8	8*16 cm	130 min	145 degrees	11 mm	7 days	No	Wound infection & disruption
9	6*15 cm	90 min	150 degrees	15 mm	5 days	No	No
10	6*12 cm	95 min	160 degrees	14 mm	6 days	No	No

Regarding flap complications, all the flaps survived well with no flap necrosis. One case had flap congestion, two cases presented with wound infection that responded to parenteral antibiotics, and one case showed wound dehiscence that healed with regular dressing and 2ry intention with no need of skin grafting to the scrotum. Regarding donor site morbidity, the donor site healed well with the scar hidden in a natural crease with accepted pliability, vascularity, thickness, and pigmentation, according to the Vancouver Scar Scale score.

With regard to the functional assessment of the testicular function, Table 3 shows semen analysis including sperm volume (ml), sperm count (million\ml), total motility (%), progressive motility (%), vitality (live spermatozoa %), and normal morphology percentage by comparing these parameters before surgery, 6 months after surgery, and 1 year after surgery. Also for hormonal analysis (testosterone, LH, FSH) in Table 4**,** there were nonsignificant changes (P≤0.005) in all parameters.

**Table 3:** Functional effects of the flap on testicular functions

Semen parameters	Preoperative	6 month postoperative	1 year postoperative
Volume (ml)	1.8 (±0.5)	1.5 (±0.45)	1.6(±0.6)
Sperm count(million\ml)	37.1 (±22.5)	33.5 (±25.33)	35.6(±23.72)
Total motility (%)	33.2 (±11.5)	31.4 (±10.3)	30.6 (±12.2)
Progressive motility (%)	26.3 (±10.55)	23.4 (±12.33)	24.8(±11.15)
Vitality (live spermatozoa %)	56(±16.8)	52.7(±13.1)	54.4 (±12.77)
Normal morphology percent %	53.2 (±16.3)	46.9 (±18.2)	50.75 (±15.83)

**Table 4:** Hormonal analysis

	Preoperative	6 monthspostoperative	1 yearpostoperative
**Testosterone(ng/ml)**	7.2±1.4	6.9±1.3	7.1±1.5
**FSH (mIU/ml)**	5.2±1.4	4.9±1.9	5.1±2.1
**LH (mIU/ml)**	3.8±1.1	3.2±1.5	3.5±0.8

Regarding patient satisfaction done by using Likert scale of 3 points (shape, irregularities, and scars) showed in Table 5**.**

**Table 5:** Likert scale of patient satisfaction

Likert scale	Very satisfied	Satisfied	Unsatisfied	Very unsatisfied
**Shape**	40%	33.33%	20%	6.67%
**Irregularities**	46.67%	33.33%	13.33%	6.67%
**Scars**	26.67%	53.33%	13.33%	6.67%

Statistical analysis: Data were analyzed by Statistical Package of Social Science (SPSS), software version 22.0 (SPSS Inc., 2013). Continuous data were expressed as Mean ± SD, while the nominal data were presented by the frequency and percentage. The one-way analysis of variance (ANOVA): Is used to determine whether there are any significant differences between the means of three independent groups. Least significance difference (LSD): It is one of the post hoc tests. It is used for multiple comparisons between groups. It was calculated at different probability values. P-value < 0.05 considered significant.

## Discussion

PostFournier's gangrene scrotal defects are often challenging for reconstructive surgeons. Even though small defects (less than 50%) are easily covered by a scrotal flap, it should be noted that if there is insufficient scrotal tissue, it requires more advanced surgical techniques to maintain the scrotum aesthetically and functionally. (11)

Although not as durable as flaps, a split-thickness skin graft may be an easier alternative for extensive scrotal defects. It should be noted that the scar contracture from a split-thickness graft can result in the loss of the cremasteric reflex, which will eventually result in the loss of the normal, testicular protective mechanisms during everyday activities. (14)

The use of myocutaneous flaps have proven beneficial due to their extensive blood supply, allowing the tissue to resist any infection, but on the downside, the flaps’ bulkiness and insulating effect elevate the temperature of the testicle, so negatively affect spermatogenesis and also badly affect cosmesis. (15)

Since 1972, researchers and doctors have studied the anatomy and functionality of the groin flap, which has been proven to be dependable as a pedicle or free flap for tissue coverage of the forearm and hand, seeing how quick and easy its harvest is and its reliable blood supply.

Perforator flaps are commonly used for the reconstruction of the abdominoperineal region; these flaps are now the first-choice treatment for soft tissue reconstruction to limit donor site sequels. The use of the propeller groin flap restored the usefulness of the groin donor site and allows great versatility in reconstruction. (1)

In the present study, 10 patients had skin loss secondary to Fournier's gangrene and trauma. All the patients were prepared by performing multiple extensive debridement and daily dressings until the scrotal wound became clean and ready for soft tissue coverage. Then, wound coverage was done using unilateral propeller groin flap for the 10 patients.

We use the flap unilaterally because the wide arc of rotation provided by the propeller technique makes the unilateral flap cover larger defects with less donor site morbidity and short operation time. The early results are excellent with good skin quality and testicular support with minimal early complications (two cases showed flap congestion, no flap necrosis, one case of wound disruption, and two cases of wound infection). All complications were controlled with conservative measures and did not affect flap survival.

The donor site morbidity was minimal with two cases of wound infection, where one of them subsided with parenteral antibiotics, and the other led to wound dehiscence that needed split thickness graft. The final appearance of the flap was both cosmetically and socially acceptable for the patient, and all patients had normal free movement of testes inside the neoscrotum.

These results are in accordance with the results of Florian B. et al., which stated that groin propeller flaps are a good choice in the reconstruction of the perineal area because the morbidity of its donor site is significantly lower than that of an abdominal flap (ex. TRAM or DIEP). Some advantages of the groin propeller flap include its naturally thin fasciocutaneous flap, the donor site has lower risk or morbidity, and it is mostly self-closing, which does not expose the structure. (16)

The size of the groin flaps varies and can be very large due to the perforating structures, which usually have an extensive course (externally, oblique, superiorly, etc.) throughout the groin, whether it being a neovascular bundle or muscle. For example, the origin of the common femoral artery allows for a more proximal repair than a surgical repair in the lower limbs (an advantage compared to ALT, which is more distal). (17, 18)

The majority of complications of the propeller groin flap were due to surgical malpractice, which included harvesting the flap incorrectly, injury to the perforating vessels, and injuiry of lateral femoral cutaneous nerves, the latter causing absence of sensation on the lateral aspect of the thigh, which may or may be fixable over time. (1)

The cushioning effect that the propeller groin flap provides for the testis has proven superior to skin grafts, especially in younger active patients. (19)

On long-term follow-up the shape, color, hang, or ptosis of the scrotum, and the patient gate looked normal as we had noticed in these patients. All our patients were psychologically satisfied regarding the reconstruction.

The dependent and external positions of the reconstructed scrotum may have similar thermoregulatory effects on the testes as that of normal scrotum, which is proven by the results of the semen analysis before surgery compared to the results 6 months and 1 year after surgery, which showed nonsignificant changes in all parameters.

Similarly, Agarwal and colleagues reported normal testicular function (using a testicular biopsy) 6 months after the implantation of the testis in the thigh. (14)

Wang et al. found that spermatogenesis was not affected in the earlier stages after the procedure but following two years, spermatogenesis was found to be abnormal. (12)

Wang et al. in another study found that spermatogenesis can be improved via thin trimming of the scrotal flap. (16)

Shen et al. showed that if you use a thin flap or place the testes just underneath the skin, it may be an alternative method for preserving spermatogenesis. (19)

In the present study, we found a nonsignificant change in the semen analysis after 6 months and one year of the surgery.

## Conclusion

**Figure 2:**
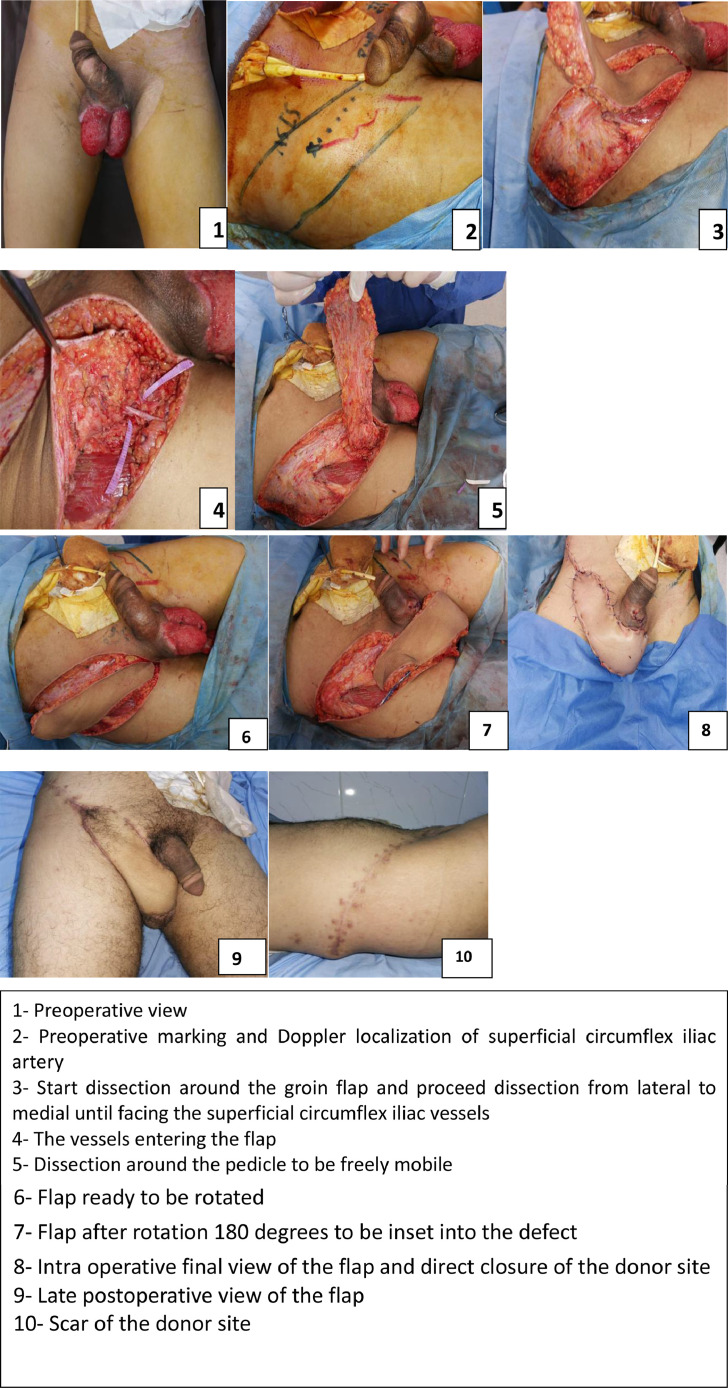
1- Preoperative view, 2- Preoperative marking and Doppler localization of superficial circumflex iliac artery, 3- Start dissection around the groin flap and proceed dissection from lateral to medial until facing the superficial circumflex iliac vessels, 4- The vessels entering the flap, 5- Dissection around the pedicle to be freely mobile, 5- Dissection around the pedicle to be freely mobile, 6- Flap ready to be rotated, 7- Flap after rotation 180 degrees to be inset into the defect, 8- Intraoperative final view of the flap and direct closure of the donor site, 9- Late postoperative view of the flap, 10- Scar of the donor site.

The use of groin flap as propeller allows for free movement and rotation of the flap, which makes the unilateral flap to cover a larger scrotal defect with good flap vascularity, lesser complications, no need for further operations to separate the pedicle, and the least donor site morbidity. This flap offers minimal donor site morbidity and minimal major complications with acceptable cosmetic outcomes (Fig. 2).

## Author Contributions

Concept -Design – operative management and technique- Supervision - Resources - Data Collection and/or Processing - Literature Search – Writing Manuscript.

## Financial support and sponsorship

Nil.

## Ethics approval

All procedures performed in studies were in accordance with the ethical standards of the institutional and/or national research committee of our institution Benha Faculty of Medicine and with the 1964 Helsinki Declaration and its later amendments or comparable ethical standards.

## Informed consent

Informed consent was obtained from all individual participants included in the study. The participant has consented to the submission of the data to the journal.

## Patient consent

Patients signed informed consent regarding publishing their data and photographs.

## Conflict of interests

Nil.
